# Comparative prevalence of hepatitis B virus infection among pregnant women accessing free maternal care in a tertiary hospital in Ghana

**DOI:** 10.1371/journal.pone.0263651

**Published:** 2022-03-04

**Authors:** Benedict Boateng Antuamwine, Eddie Delali Herchel, Eric Mishio Bawa

**Affiliations:** Department of Biomedical Laboratory Sciences, School of Allied Health Sciences, University for Development Studies, Tamale, Ghana; Centre de Recherche en Cancerologie de Lyon, FRANCE

## Abstract

Hepatitis B virus infection is endemic in sub-Saharan Africa, and accounts for a significant proportion of morbidities and mortalities in Ghana. Infection with HBV during pregnancy can result in life-threatening complications to both mother and child. To improve their quality of life, the free maternal care was introduced to grant pregnant women cost-free access to antenatal and postnatal services. The study analysed the prevalence of HBV infection among pregnant women receiving free antenatal care in a tertiary hospital in Ghana. This was a retrospective cross-sectional study, where secondary data of pregnant women who accessed free antenatal services at the Trafalga hospital, Ho, from 2016 to 2017 were retrieved from the hospital’s database. Data on hepatitis B surface antigen reactivity test, age and period of turnout were analysed with Microsoft Excel and Graph pad prism version 6. A total of 2,634 pregnant women assessed free antenatal care from January 2016 –December 2017, with 10% rise in turnout in 2017. The age of the study population was fairly young, ranging from 13–52 years and mean of 29.87±5.83. The prevalence of HBV infection among pregnant women in the entire study was estimated to be 6.0%, while that of 2016 and 2017 were 5.3% and 6.7% respectively. Turnout for antenatal services peaked in July, which also recorded the highest prevalence of HBV infection among the pregnant women. Our study, first of its kind show an HBV prevalence of 6.0% among a large population of pregnant women who accessed free antenatal services at a tertiary hospital in Ghana. The study evaluates the influence of the free maternal care policy on antenatal attendance and HBV infection rates among pregnant women.

## Background

The free maternal healthcare policy adopted by Ghana in 2008 is an absolute requisite intervention that provides to pregnant mothers free and easy access to healthcare delivery in all public and selected private healthcare facilities across the country [1]. This has enhanced the routine turnouts of pregnant mothers requiring antenatal assessment, especially in rural communities where antenatal visits aren’t encouraging [2, 3].

The major goal for rolling out such policy was towards reducing maternal and infant mortality by providing to all, convenient, reliable and unlimited access to antenatal and postnatal care services [4, 5]. Under these services, pregnant mothers undergo several compulsory clinical assessments that promote the wellbeing of the mother and the unborn baby. Among these clinical assessments also includes laboratory testing for infectious pathogens, of which screening for hepatitis B virus (HBV) is of high relevance [6, 7].

HBV infection is a global public health issue, affecting more people than HIV [8] and ranked the 7^th^ leading cause of mortality worldwide in 2013 [9]. Despite the availability of safe and effective vaccines since the 1980s [10], the availability of successful treatment since 1991 [11], and the implementation of universal vaccination programs [11], HBV is still endemic in sub-Saharan Africa, with an estimated seroprevalence of 6.1% [12]. As of 2015, 257 million people were living with chronic HBV infection [13].

In Ghana, this situation is not any different, where HBV remains a huge public health issue [14]. Although there is currently the availability of a safe and effective vaccine, Ghana is considered an endemic zone [15] with a prevalence of 8.36% among adults, 14.30% in the adolescent population and 0.55% in children less than 5 years old [14]. The prevalence varies regionally from 4.8% to 12.3% in the general population, 10.8% to 12.7% in blood donors and about 10.6% in pregnant women [16]. Ghana is therefore among the areas within sub-Saharan Africa considered to be highly endemic for HBV infection [17].

Calls for interventions on reducing the risk of HBV transmission saw the birth of a number of policies, including the national policy on viral hepatitis [6], national health policy [18], technical guidelines for integrated disease surveillance and response in Ghana [19] the expanded programme on immunization (EPI) [20] and the national health insurance scheme (NHIS) [21] among others. Undoubtedly, the strategies have contributed to reduction in the incidence of new infections nationwide, even though a lot more can be achieved.

For example, the NHIS introduced in 2003, which also included the reputable free maternal care for pregnant women in 2008, improved overall access to health services by eliminating financial barriers to healthcare delivery. Under this scheme, only pregnant women and infants receive free screening for HBV, with infants further vaccinated against the infection under the EPI. On the other hand, screening for others are only covered under the scheme when they are prescribed at hospitals for patients “suspected” to be reactive to HBV. Additionally, hepatitis B immunoglobulin G and hepatitis B monovalent vaccine for babies born to hepatitis B reactive mothers are also not covered by the NHIS [15].

Viral hepatitis during pregnancy is associated with a high risk of maternal complications. The infection adversely affect pregnancy outcome, leading to spontaneous abortion, premature delivery, intrauterine growth restrictions, and low birth weight of infants [22]. In addition, the high rate of vertical transmission of the virus to the unborn child during delivery, referred to as mother to child transmission (MTCT) could impact the child’s quality of life. Fetal and neonatal hepatitis impairs the physical and mental development of infants [23].

In endemic areas, such as Ghana, HBV infection among children occur mainly during infancy and early childhood, with MTCT accounting for approximately half of the transmission routes of chronic HBV infections [16, 24]. Prevention of MTCT therefore remains an essential step in reducing the global burden of chronic HBV in children. Largely, cases of MTCT of HBV occur at birth, and therefore providing immunoprophylaxis to newborns is an excellent way to block natal transmission. The WHO 2030 goal of HBV elimination could only come to realization when full coverage of the universal HBV vaccination and HBV-birth dose prophylaxis is prioritized in sub-Saharan Africa [12]. Without prophylaxis, a mother who is positive for hepatitis B surface antigen (HBsAg) confers up to 90% risk of passing the infection to her offspring [25]. Consequently, it is imperative to screen and identify infected mothers whose babies will benefit from HBV immunoprophylaxis while vaccination against the virus in neonates born to HBV negative mothers will be a cost-effective measure towards eradication of the infection.

Ghana introduced vaccination against hepatitis B in infants as part of the EPI in 2002 [26]. After 6 weeks of life, infants are administered with the pentavalent vaccine to build immunity against the infections; hepatitis B, diphtheria, polio, tetanus, and influenza type B [15]. However, this vaccine provides immunity against HBV infection in infants born to HBsAg negative mothers but does not prevent perinatal hepatitis B infection. To effectively prevent MTCT of hepatitis B, the single-dose hepatitis B vaccine or immunoprophylaxis must be administered within 12 to 24 hours of birth [27].

Undoubtedly, the several strategies adopted to slow down the transmission of HBV among groups of individuals across the country look promising. Yet, data on the positivity rate, especially among pregnant mothers within the Volta region of Ghana are limiting. Our study, first of its kind evaluates the turnout and HBV infection rate among pregnant mothers accessing free ANC at the Trafalga hospital of the Volta region. Boasting of a large sample size in our study, we show here an increased turnout of pregnant mothers accessing ANC with considerably reduced prevalence of HBV infection. The study provides ample amount of data on HBV infection in pregnancy and will be crucial in the formulation of policies aimed at improving maternal and child health.

## Materials and methods

The study was approved by the Department of Biomedical Science, UDS and by the School of Allied Health Sciences Ethical Review Committee. Appropriate permission was granted by the management of the Trafalga hospital who maintained ethical standards and obtained patients’ consent in the collection, handling and storage of patients data for the purposes of research. Data collected was kept confidential and password protected.

### Study design and area

This was a retrospective study conducted at the Trafalga Hospital, located in Ho, the capital of the Volta Region of Ghana. The hospital currently serves as the regional hospital and a referral facility for the district hospitals in the Volta Region. It has a wide coverage that spans the entirety of the region and even includes some communities in neighbouring Togo.

### Data collection

Secondary data of pregnant women who accessed ANC services at the Trafalga hospital from the start of 2016 and to the end of 2017 were retrieved from the Hospital Administration and Management database. This was done in mid-year of 2018 after a data request form was completed and approved by the hospital authority. Data was anonymized before collection and included the age, date of visit and HBsAg test results of patients. Authors had no access to information that could identify individual patients.

### Data management and statistical analysis

To eliminate bias, duplicates and incomplete data were eliminated. Data finally analysed consisted of first timers at the ANC unit within the 2-year period. Analysis of data was aided by Microsoft Excel and Graphpad Prism version 6. Data are presented as means, frequencies and graphs. Unpaired t-test was used for comparing continuous variables whiles the Fisher exact test was used for comparing categorical values. A p-value of less than 0.05 was considered statistically significant.

## Results

A total of 2,634 pregnant women assessed free antenatal care from January 2016 –December 2017. The age of the study population was fairly young, ranging from 13–52 years and with a mean of 29.87±5.83. The turnout of pregnant women in 2017 at the facility was 10% higher but slightly younger (29.87±5.83) compared to those who visited the facility in 2016 (30.12±5.82) (p = 0.0392) as shown in Table 1.

**Table 1:** The hepatitis B reactivity status and age distribution of pregnant women accessing free antenatal care.

	2016 (N = 1254)			2017 (N = 1380)			TOTAL (N = 2634)		
Age	Positive	Negative	Total	Positive	Negative	Total	Positive	Negative	OVERALL TOTAL
Mean	30.74±5.67	30.08±5.83	30.12±5.82*	29.58±5.63	29.65±5.84	29.65±5.83*	30.06±5.66	29.86±5.84	**29.87±5.83**
Range	19–43	14–50	14–50	15–40	13–52	13–52	15–43	13–52	13–52
< 20	2(0.2%)	46(3.7%)	48(3.8%)	6(0.4%)	62(4.5%)	68(4.9%)	8(0.3%)	108(4.1%)	116(4.4%)
20–29	24(1.9%)	493(39.3%)	517(41.2%)	38(2.8%)	554(40.2%)	592(42.9%)	62(2.4%)	1047(39.8%)	1109(42.1%)
30–39	35(2.8%)	577(46.0%)	612(48.8%)	46(3.3%)	625(45.3%)	671(48.6%)	81(3.1%)	1202(45.6%)	1283(48.7%)
40–45	5(0.4%)	66(5.3%)	71(5.7%)	2(0.2%)	43(3.1%)	45(3.3%)	7(0.3%)	109(4.1%)	116(4.4%)
˃ 45	0(0.0%)	6(0.5%)	6(0.5%)	0(0.0%)	4(0.3%)	4(0.3%)	0(0.0%)	10(0.4%)	10(0.4%)
Total	**66(5.3%)**	1188(94.7%)	1254(100.0%)	**92(6.7%)**	1288(93.3%)	1380(100.0%)	**158(6.0%)**	2476(94.0%)	2634(100.0%)

Continuous data are presented as mean±SD while categorical data are presented as frequencies and percentages. Comparisons between continuous data was made employing unpaired student t-test while categorical data was compared using fisher exact test. Statistical significance was set at; *p<0.05.

Majority of the pregnant women fell within the age bracket 30–39 years, making up 48.7% of the entire study population and registering the highest prevalence of hepatitis B infection, which was closely followed by those in the age group 20–29 years. It is worth noting that teenage pregnancy contributed to 4.4% of the study population with a hepatitis B prevalence rate of 0.3% (Table 1).

**Fig 1:**
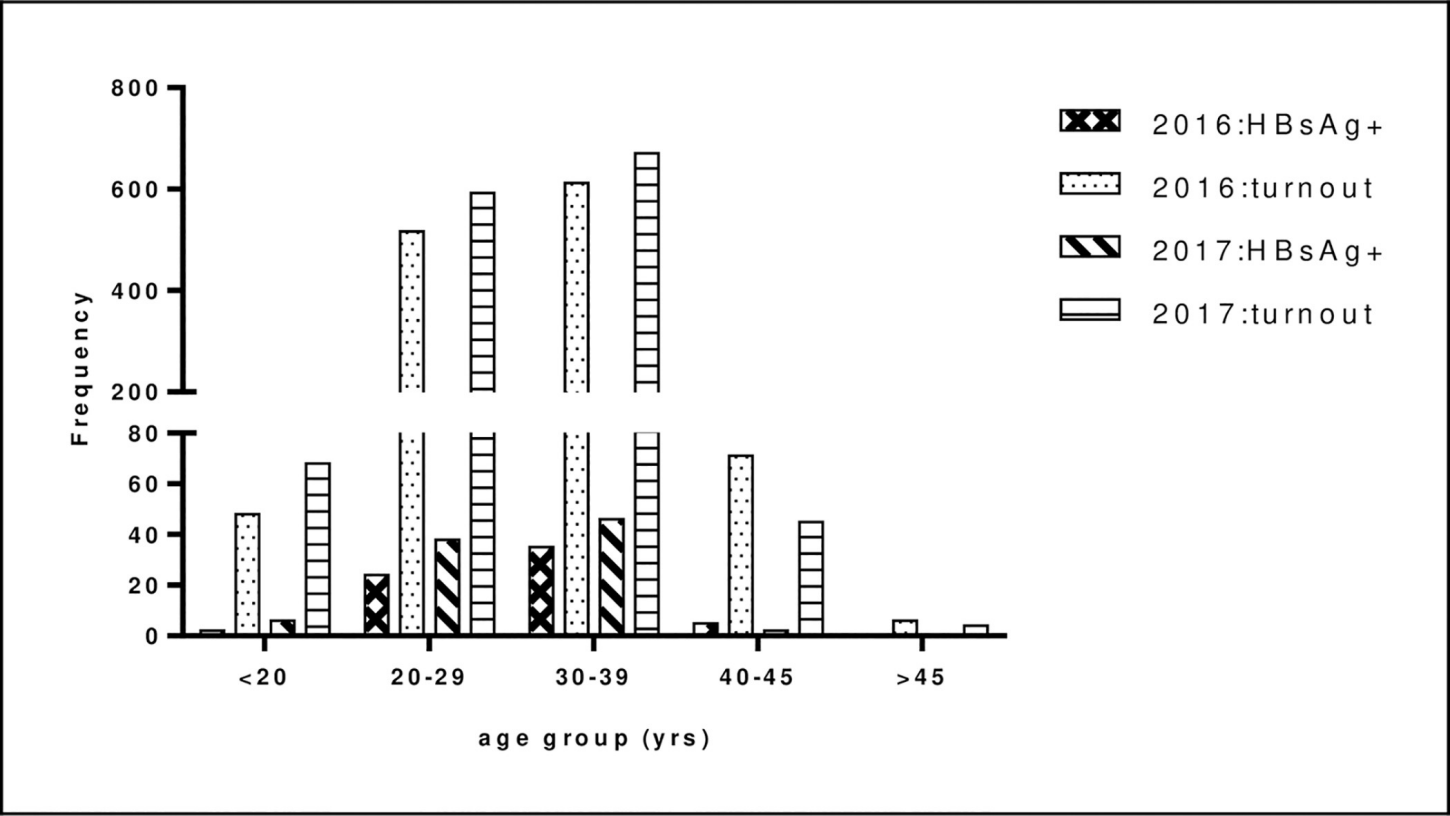
Frequency distribution and hepatitis B prevalence among pregnant accessing free antenatal care stratified by age group.

The prevalence rate of hepatitis B infection was observed to increase with an increasing age group of pregnant women, peaking at the 30–39 years age group and then declining sharply in older groups (Fig 1). Interestingly, the turnout of pregnant women within each age group at the facility was observed to determine their respective prevalence rates, with increasing turnouts providing higher rates as shown in Fig 1. This trend is further observed in the entire yearly visits, where a 6.7% hepatitis B prevalence rate was determined among 1,380 pregnant women accessing the ANC facility in 2017 compared to the 5.3% prevalence rate determined among the 1,254 pregnant women who visited the facility in 2016.

**Fig 2:**
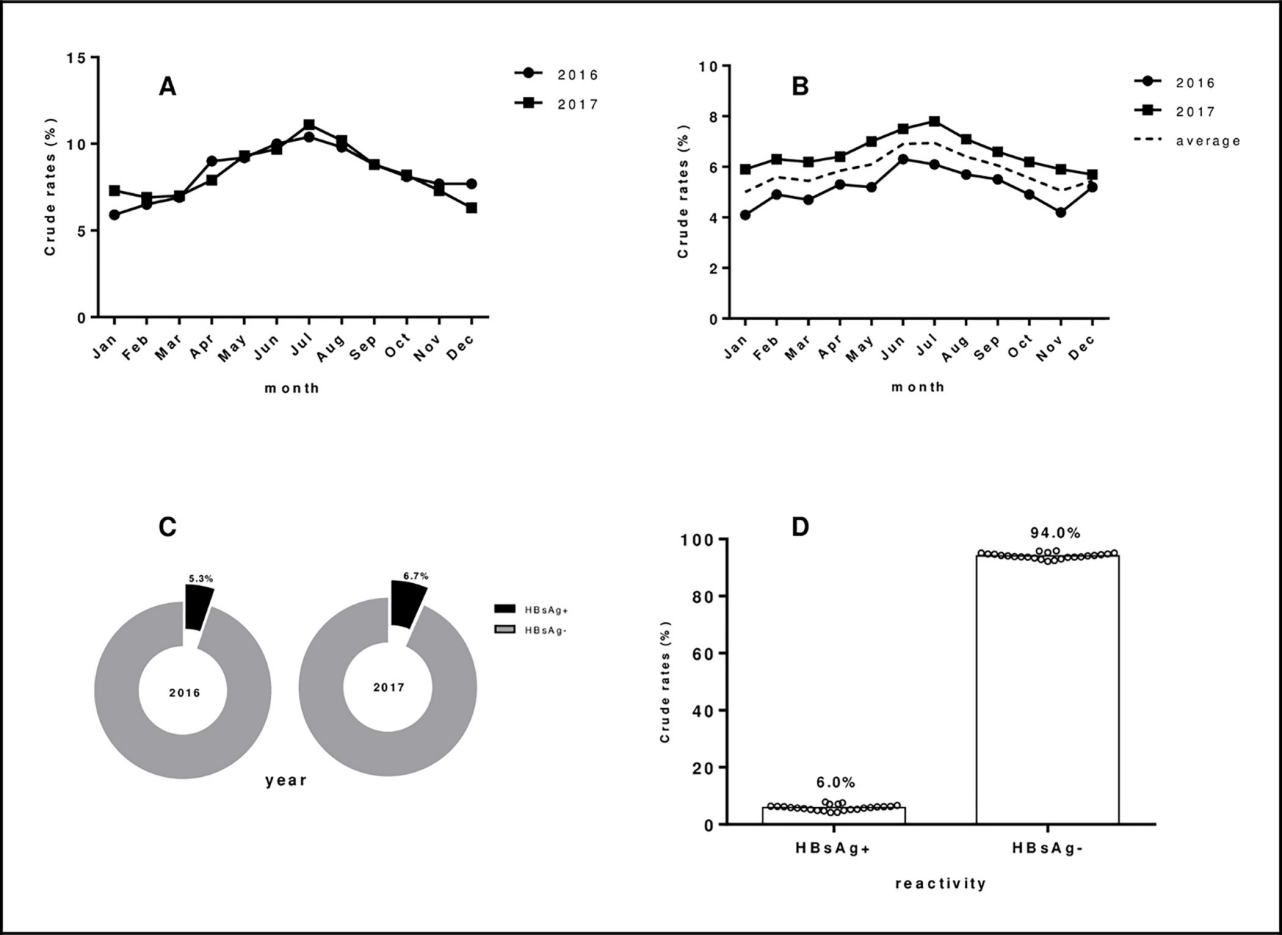
Hepatitis B prevalence and turnout rate of pregnant mothers accessing free maternal care stratified according to month and year. A; monthly turnout rates in 2016 and 2017 B; monthly prevalence rates of hepatitis B in 2016 and 2017 C; prevalence rates of hepatitis B in 2016 and 2017 D; overall prevalence of hepatitis B in the study. HBsAg+/-; positive/negative for hepatitis B surface antigen.

Considering the high turnout of pregnant women who accessed maternal healthcare at the facility from the beginning of 2016 to the close of 2017, we evaluated whether their turnouts at the facility could have been influenced by seasonal changes of the year. The monthly turnout rates of pregnant women for year at the facility were hence determined. A gradual increase in the monthly rate of turnout was observed from January, plateauing at the months of June, July, August and then declining in the last quarter of the year as shown in Fig 2A. This trend was unchanged between 2016 and 2017. The least numbers of pregnant women who visited the facility were recored at the beginning (January, February) and end (November, December) of the year. Seasonal changes could therefore be a determining factor for the numbers of pregnant women who turned out at the facility.

A similar trend was also observed in the monthly prevalence of hepatitis B (Fig 2B), probably indicative of a direct proportionality between turnout and prevalence of hepatitis B infection. The highest average monthly prevalence of hepatitis B infection among pregnant women was observed in July followed by June and then August while the least prevalence rate was observed in January. When the yearly prevalence was estimated, it was noted that the prevalence of the infection among pregnant mothers in the Volta region was higher in 2017 (6.7%) compared to 2016 (5.3%) as shown in Fig 2C. This further strengthened the notion that the more pregnant women turnout at the facility, the more likelihood of detecting many cases of pregnant mothers testing positive for HBsAg, as more pregnant mothers (1380) visited the ANC in 2017 compared to that of 2016 (1254).

The overall prevalence of HBV infection among pregnant women in the study was estimated to be 6.0%, shown in Fig 2D. This represented 158 pregnant mothers who tested positive for the infection at the ANC facility in the period of 2 years.

## Discussion

We studied the trend of HBV infection among pregnant women accessing free maternal care from 2016 to 2017. Our study estimated the prevalence of hepatitis B among pregnant women accessing free ANC at the largest hospital in the Voltal region to be 6.0%. In comparison with similar but much earlier studies across the country, where higher prevalences of 16.0%, 12.3%, 10.6% and 10.5% were reported [15, 28–30], we observe in our study a relatively lower prevalence of HBV infection among pregnant women. Measures such as the free maternal and child care, EPI, increased surveillance on viral hepatitis infection and increased health education adopted over a decade ago could potentially have contributed to significant drops in the national prevalence of hepatitis B, and hence among pregnant women as well. A recent study in the Northern region of Ghana also reported a HBV prevalence among pregnant women to be 7.9% [31] compared to the much higher prevalences recorded in earlier studies. In addition, the Volta region could potentially be the region with very few cases of hepatitis B infection, possibly the reason why the region has limited number of studies on hepatitis B infection. Therefore the findings of this study may be the true picture of HBV infection among pregnant women in the region. In neighbouring Nigeria, a prevalence of 7.9% was estimated among pregnant women [32].

The highest prevalence of hepatitis B among the pregnant women in this study was recorded in the age bracket 30–39 (3.1%) followed by 19–29 (2.4%). It was least common among teenage mothers and completely unassociated with pregnant mothers beyond 45 years. The high prevalence among the 30–39 age bracket and that of the 19–29 compared to the other age brackets could be associated with engagement in sexual activity. These age groups fall within the sexually active age brackets, hence are at a higher risk of HBV infection compared to those engaging in less sexual activity, since sex is one of the major routes of HBV transmission. Similar findings were made by Luka and colleagues [33] in Nigeria, where they recorded the highest prevalence of hepatitis B among the 30–34 age bracket followed by the 25–29 age bracket and the least in the 20–24 age bracket. It is worth mentioning that teenage pregnancy is still a major challenge in Ghana [34]. It is therefore not unsual to record 4.4% of pregnant mothers to be under 20 years of age, with as young as 13 year old pregnant mothers. The prevalence of adolescent pregnancy in Africa was estimated to be 18.8% and within the sub-Saharan African region, 19.3% [35].

When the data of 2016 and 2017 were compared, there was an increased turnout of pregnant women at the facility in 2017, who in turn were slightly younger. This may have accounted for the higher prevalence of hepatitis B infection among pregnant women recorded in 2017. As a sexually transmitted disease [36], HBV is likely to be common among younger women, who are often sexually active. The prevalence of hepatitis B increased from 5.3% in 2016 to 6.7% in 2017. The sharp increase in the prevalence maybe because there was an increase in turnout. The turnout of pregnant women increased from 47.6% in 2016 to 52.4% in 2017. The high turnout of pregnant women is probably due to the on-going advocacy on the several national programs on health, such as the free maternal care policy in the country aimed at promoting maternal and child health.

Analysis of the monthly turnout of pregnant women at the facility was shown to peak in July irrespective of the year, which also recorded the highest monthly prevalence (6.9%) of HBV infection. The highest rainfall patterns in Ghana are recorded in the month of June–July, which comes with increased malaria infection [37]. Therefore, the likelihood of malaria infection among pregnant mothers during this period is high, and may have compelled them to seek medical attention at the ANC facility of the Trafalga hospital. Low cases of malaria are usually recorded from November–February, which further supports the low turnout during these months.

Though the prevalence of hepatitis B among the pregnant women as found out in this study was low, we encourage the vaccination of potential mothers against HBV. Vaccination against hepatitis B is not part of the free maternal healthcare policy. Aside from targeting the neonates for vaccination, pregnant women who test negative should be vaccinated after delivery. This measure can result in a further reduction of the prevalence of hepatitis B. There is, therefore the need to campaign for HBV vaccination among mothers after delivery.

Informed by global goals and targets, there is the need for countries to develop practicable national policies such as vaccination of pregnant women and mothers to halt the transmission and further spread of the hepatitis viral infection. These measures should be carried out taking into consideration the country’s viral hepatitis prevalence, populations affected, structure and capacity of the healthcare and community systems, and resources that can be mobilized towards achieving these global targets.

While the present study prides itself in its large sample size of expectant mothers accessing antenatal care and outcomes of their hepatitis B screening test over 2 years, some limitations may be apparent. Records on the localities of subjects are lacking. This does not allow for the clustering of subjects based on their areas of residence. Hence, the possibility of timely identifying zones within the study setting that may be hotspots of HBV infections or teenage pregnancies could almost be uncertain. Additionally, the fact that these expectant mothers could present with comorbidities including malaria, HIV, hypertension, and diabetes among others at the time of the visit, indicates the relevance of collecting data on these variables. This would have strengthened the data repository of our study while encouraging a more robust interpretation. Despite the study being entirely a retrospective assessment of the antenatal visits of expectant mothers, a prospective study in parallel to investigate the likelihood of mother-to-child transmission of HBV infection would have been complementary. The study did not also segregate between subjects who were vaccinated against HBV infection from those who were not. This is significant in evaluating the rate of hepatitis B vaccination among women within the region in order to make informed decisions and for the purposes of planning.

## Conclusion

Generally, there was an increase in the turnout of pregnant women at the ANC facility of the Trafalga hospital from 2016 to 2017. The prevalence of hepatitis B among pregnant women was found to be 6.0%, with the highest monthly prevalence of 6.9% recorded in July. Our study draws attention to the need to institute and strengthen policies that will encourage hepatitis B vaccination especially among women of reproductive age. There is also the need to include hepatitis B vaccination in the free maternal care policy to cater for mothers who are qualified to take the vaccine.

## Supporting information

S1 Checklist PLOS one clinical studies checklist. (PDF)

S1 Data (ODS)

## Abbreviations

ANC: antenatal care
HBV: hepatitis B virus
MTCT: mother-to-child transmission
EPI: expanded programme on immunization
NHIS: national health insurance scheme
PNC: postnatal care
